# MAMPI-UWB—Multipath-Assisted Device-Free Localization with Magnitude and Phase Information with UWB Transceivers †

**DOI:** 10.3390/s20247090

**Published:** 2020-12-10

**Authors:** Marco Cimdins, Sven Ole Schmidt, Horst Hellbrück

**Affiliations:** 1 Technische Hochschule Lübeck, University of Applied Sciences, 23562 Lübeck, Germany; sven.ole.schmidt@th-luebeck.de (S.O.S.); horst.hellbrueck@th-luebeck.de (H.H.); 2 Institute of Telematics, University of Luebeck, 23562 Lübeck, Germany

**Keywords:** device-free localization, multipath component, channel impulse response, feature vector, error model, position error probability, ultra-wideband, knife-edge diffraction

## Abstract

In this paper, we propose a multipath-assisted device-free localization (DFL) system that includes magnitude and phase information (MAMPI). The DFL system employs ultra-wideband (UWB) channel impulse response (CIR) measurements, enabling the extraction of several multipath components (MPCs) and thereby benefits from multipath propagation. We propose a radio propagation model that calculates the effect on the received signal based on the position of a person within a target area. Additionally, we propose a validated error model for the measurements and explain the creation of different feature vectors and extraction of the MPCs from Decawave DW1000 CIR measurements. We evaluate the system via simulations of the position error probability and a measurement setup in an indoor scenario. We compare the performance of MAMPI to a conventional DFL system based on four sensor nodes that measures radio signal strength values. The combination of the magnitude and phase differences for the feature vectors results in a position error probability that is comparable to a conventional system but requires only two sensor nodes.

## 1. Introduction

In recent decades, radio-frequency communication has entered various industrial and commercial applications. Some examples are WLAN replaced Ethernet cables. Bluetooth connects wearables such as headphones and smartwatches. RFID is widely included in systems to track goods and replaces barcodes in retail. GPS is commonly applied for outdoor positioning.

There has been a noteworthy increase in wireless technologies in an industrial context. Other technologies aim to create wireless sensor networks based on battery-driven sensors. IEEE 802.15.4, ZigBee, and LoRaWAN are technologies that operate in license-free ISM bands. During operation, those sensors and devices exchange messages via radio-frequency transceivers. On reception, devices measure channel parameters such as received signal strength (RSS), channel state information (CSI), or channel impulse responses (CIR) at the physical layer of the radio-frequency chips. Such channel parameters are used in routing protocols that depend on link-quality assessments. Additionally, device-free localization (DFL) systems apply those measurements to detect and track anomalies, such as moving persons in direct proximity.

Typical applications for device-free localization are elderly care [1], intrusion detection, crowd estimations [2], and smart-home applications. Therefore, device-free localization systems monitor the alteration of the radio-frequency channel by accessing the above-mentioned channel measurements. In contrast to camera-based systems, device-free localization preserves privacy and may work in non-line-of-sights conditions as well as in darkness and smoke or with obstructed sensors. An overview of radio-frequency-based device-free localization systems is given in [3].

Device-free localization has been the focus of research for more than a decade [4]; however, there are still major challenges to be solved. One major challenge is the number of sensors to cover the target area. In particular, when existing wireless devices serve as wireless sensor nodes for DFL, we cannot expect many sensors within a room. In addition, many wireless sensors increase the installation and maintenance costs and affect the acceptance of the system negatively. Another major challenge is the presence of multipath propagation. In narrowband radio-frequency systems, multiple signal paths overlap at the receiver causing short-term fading. An advantage is that radio signals are received even though there is no direct line-of-sight condition. The difficulty is to differentiate whether the observed short-term fading is caused by a change in the environment or by the presence of a new obstacle, e.g., a person that we aim to localize.

**Figure 1:**
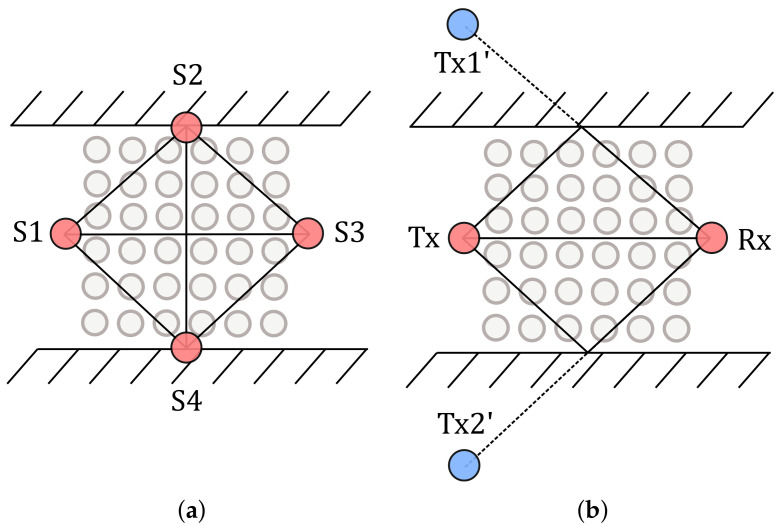
Comparison of DFL systems ©2020 IEEE, Reprinted, with permission, from [5]. (**a**) Conventional DFL. (**b**) Multipath-assisted DFL.

Figure 1a shows a conventional device-free localization system that monitors a corridor. The four red dots symbolize sensors that span radio-frequency links between each other. In contrast to the conventional device-free localization system, we aim to create a device-free localization system that analyses the direct link between the sensors, as well as the reflection paths at the walls (Figure 1b). The multipath-assisted device-free localization system requires two physical sensors shown as red dots. By mirroring the transmitter (Tx) at the wall, we benefit from virtual transmitters (Tx’) depicted as blue dots. We use virtual sensors to determine the reflection paths for modeling.

We propose to measure the channel impulse response upon message reception with ultra-wideband radio-frequency chips. From the channel impulse response, we detect the corresponding reflections and extract the magnitude and phase. In recent years, research on indoor localization systems based on IEEE 802.15.4a has resulted in commercially available systems. Furthermore, ultra-wideband radio-frequency chips are integrated into commercially available smart devices e.g., phones and watches. Therefore, we expect that ultra-wideband radio-frequency communication will be available on many devices in the future.

In previous work, we have shown how to extract the magnitude and the phase for each unique multipath component from a channel impulse response. The response is measured by an off-the-shelf IEEE 802.15.4a compliant ultra-wideband transceiver: the Decawave DW1000 [6]. In this work, we extend our previous results and propose a multipath-assisted DFL system that is based on magnitude and phase measurements of a single sensor pair.

Our contributions are as follows:

- We design a multipath-assisted device-free localization system based on magnitude and phase measurements of multipath components.
- We derive the position error probability as a metric to compare the performance of the system before deployment.
- We refine the signal processing for extraction of magnitude and phase values from channel impulse response measurements.
- We compare the performance of a conventional DFL system to our proposed multipath-assisted DFL system using different feature vectors.

The rest of this paper is structured as follows: In Section 2, we provide an overview of DFL systems. Section 3 presents the multipath-assisted DFL system. In Section 4, we provide implementation details of our simulation and measurement setup that we evaluate in Section 5. We conclude this paper and give an outlook for future work in Section 6.

## 2. Related Work

Research of device-free localization systems started in 2007 with a paper by Youssef et al., providing the proof-of-concept of a device-free localization system and providing challenges and future research directions [4]. Several authors demonstrated in tag-based localization systems that considering multipath components reduces the number of nodes or that a single anchor is enough to cover the target area [7,8,9]. Similarly, we aim to turn multipath propagation for device-free localization from foe to friend.

There have been several device-free localization systems with UWB signals developed in the past. Kilic et al. designed a DFL system that detects and localizes persons by exploiting frequency variations in the time domain [10]. Wang et al. propose UWB sensors that measure the received signal strength variation based on CIR measurements for device-free localization [11]. In [12], Bregar et al. deploy a radio tomographic imaging-based DFL system with UWB radios. They measure the distance from the nodes with time-of-flight measurements and the DFL system determines the value of the first CIR peak. In comparison to those approaches, we propose a DFL system that measures the UWB CIR in a first step and, in a second step, extracts the complex signal components from each MPC. Schmidhammer et al. have shown previously how to extract multipath components from UWB CIR measurements [13,14]. In addition, they demonstrated that they can detect vehicles with channel sounders [14]. In [15], Ledergerber et al. present a multi-static radar solution based on UWB transceivers. To localize the person, they inspect the mean and the variance of the measured CIR and determine the position of a person by a particle filter.

In [16], Unterhuber et al. present the idea of including multipath-assisted device-free localization as an application scenario called Wi-Fi Sensing in future IEEE 802.11 standards.

Gunia et al. demonstrated that hybrid localization systems inherit lower localization errors. Hybrid localization systems do not rely on a single type of distance measurements, such as the received signal strength or time-of-flight, or time-difference-of-arrival, but a combination of such approaches [17]. Therefore, we investigate whether hybrid feature vectors combined of magnitude and phase measurements improve device-free localization system.

Our goal is to develop a model-based device-free localization system that is free from extensive training periods. In [18], we model the impact of a person with a knife-edge diffraction model. Although the model has been derived for narrowband radio-frequency signals, we applied the model to the magnitude and phase values that are extracted from multipath components of CIR measurements [6]. In addition, Schmidhammer et al. proposed a more complex radio propagation model for the calculation of the magnitude of the multipath components [19]. As our proposed radio propagation model, their model is based on knife-edge diffraction. Their model calculates the effect of the magnitude on the projection of the 3-dimensional obstacle and was tested with a channel sounder.

In this paper, we derive the position error probability as a metric for an intuitive approach for accessing the performance of a multipath-assisted DFL system prior to deployment. We determine whether the feature vector at a position can be distinguished from the noise of the received signal. Other approaches include the Cramer-Rao Lower Bound, as shown by Rampa et al. with a diffraction model of a narrowband DFL system in [20]. With the position error probability, we will compare a multipath-assisted device-free localization system that is composed of different feature vectors to a conventional device-free localization system. Furthermore, we will compare the application of the radio propagation model in a test setup and compare the simulated to measured values. Instead of using channel sounders, our system uses commercially available off-the-shelf UWB transceiver the DW1000 from Decawave. In contrast to our previous work in [6], we refine the signal processing to extract the multipath components from the CIR measurement.

## 3. MAMPI DFL Approach

In this section, we design our multipath-assisted DFL system with magnitude and phase information (MAMPI). In Section 3.1, we explain the underlying radio propagation model to calculate the impact on the received signal due to the position and diameter of the person. To prove the concept of our DFL system, we implement a simple fingerprinting approach that is described in Section 3.2. The received signal has magnitude and phase information, therefore, we design different feature vectors that are investigated in this paper in Section 3.3. Finally, in Section 3.4, we derive the error model to calculate the performance of the system prior to deployment.

### 3.1. Radio Propagation Model

In this section, we present the radio propagation model that determines the change in the magnitude and phase based on the position and diameter of the person.

**Figure 2:**
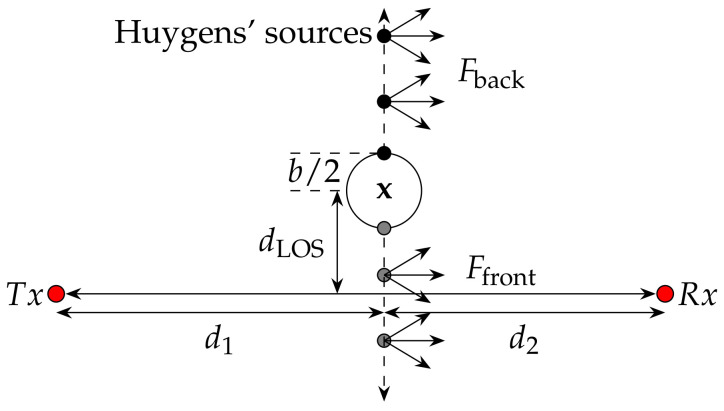
Distances and resulting Fresnel integrals that are required by the radio propagation model ©2019 IEEE, Reprinted, with permission, from [6].

The knife-edge diffraction-based model calculates the effects of an obstacle on the received radio-frequency signal. Typically, we assume that the transmitter emits a wave in the far-field of the transmitter. The far-field for a center frequency of 4.5 GHz starts after approx. 2·λ=13 cm. Following the knife-edge diffraction, the wavefront does not penetrate an obstacle. Instead, at the edges, the wavefront resolves into Huygens’ sources that will form a new wavefront, which propagates to the receiver [21]. Figure 2 shows a typical scenario. For more details on this model, we refer to our previous publication in [18].

To apply this model, we require the positions of the transmitter Tx and the receiver Rx, as well as the position x and diameter *b* of the person. Based on those three positions, we calculate the distance $d_{1}$ between Tx and the height of x and the distance $d_{2}$, which is the distance from the height of x towards Rx. $d_{\mathrm{LOS}}$ is the shortest distance between the position x and the line between Tx and Rx, which is following the direction of $d_{1}$ and $d_{2}$. With the known distances and the wavelength λ, we calculate the Fresnel–Kirchoff parameter *v*:

(1) $$v=d_{\mathrm{LOS}}\sqrt{\frac{2(d_{1}+d_{2})}{\lambda d_{1}d_{2}}}.$$

When the target area is vacant, the receiver Rx measures the electromagnetic free space field strength $E_{0}$. Later, the free space field is measured during an idle phase where the target area is vacant from persons. When a person enters the scene, Rx senses an altered field strength *E* relative to the free space field $E_{0}$. The complex Fresnel integral F(v) is the ratio $E/E_{0}$ describing the impact of the person [21]:

(2) $$F\left(v\right)=\frac{E}{E_{0}}=\frac{1+j}{2}\left[\left(\frac{1}{2}-C\left(v\right)\right)-j\left(\frac{1}{2}-S\left(v\right)\right)\right],$$

with S(v) and C(v) being the Fresnel sine and cosine, respectively, which are solved numerically.

As shown in Figure 2, the person standing at position x has a certain diameter *b*. We calculate two Fresnel integrals to determine its impact on the signal. The first integral $F_{\mathrm{back}}$ describes the Huygens’ sources from the back of the person towards infinity. The second integral $F_{\mathrm{front}}$ describes the Huygens’ sources from the front of the person towards minus infinity. Therefore, we initialize the two distances $d_{LOS_{\mathrm{back}}}=d_{LOS}+b/2$ and $d_{LOS_{\mathrm{front}}}=d_{LOS}-b/2$, which result in $F_{\mathrm{front}}\left(-v_{\mathrm{front}}\right)$ and $F_{\mathrm{back}}\left(v_{\mathrm{back}}\right)$, respectively. The overall influence of the person on the received signal is the superposition of both values:

(3) $$F_{\mathrm{person}}=F_{\mathrm{front}}\left(-v_{\mathrm{front}}\right)+F_{\mathrm{back}}\left(v_{\mathrm{back}}\right).$$

Following Equation (3), $F_{\mathrm{person}}$ is a complex value, which enables separation in magnitude $s_{\mathrm{person}}\left(\right|F_{\mathrm{person}}\left|\right)$ and phase information $\phi_{F}$ by:

(4) $$s_{\mathrm{person}}=20\cdot log(|F_{\mathrm{person}}\left|\right)\;\left[\mathrm{dB}\right].$$

(5) $$\phi_{F}=\arctan\left(\frac{I\{F_{\mathrm{person}}\}}{R\{F_{\mathrm{person}}\}}\right)\left[\mathrm{rad}\right],$$

where I{·} is the imaginary and R{·} the real part of Equation (3).

In the following, this model is applied to predict the effect of diffraction by our target for magnitude and phase variations.

### 3.2. Position Estimation

In this section, we describe the position estimation method applied in our localization system.

We assume a target area that consists of *P* positions. Each position in the target area is referenced by its position index *r*. For each of the person’s position $p_{r}$ in the target area with r=1,...,P, we determine a reference feature vector $r_{p_{r}}$ that serves as a fingerprint and includes values to characterize $p_{r}$. Also, an observation feature vector $r_{p_{o}}$ is generated, by measuring the same parameters for the area containing a person of unknown position.

To localize this person, we check the similarity of $r_{p_{o}}$ to all reference feature vectors $r_{p_{r}}$ by calculation of the distance between the vectors. In the following, we apply the $\ell_{1}$-norm $d_{\ell_{1}}$ that is also known as the Manhattan distance:

(6) $$d_{\ell_{1}}\left(r_{p_{o}},r_{p_{r}}\right)=\sum_{l=1}^{L_{s}}\left|r_{p_{o},l}-r_{p_{r},l}\right|.$$

To estimate the position, we choose the likeliest reference feature vector $\hat{r}$, by finding the minimum of the previously calculated $\ell_{1}$-distances $d_{\ell_{1}}$.

(7) $$\hat{r}=\arg\;\min_{r}\left(d_{\ell_{1}}\left(r_{p_{o}},r_{p_{r}}\right)\right).$$

The position related to the closest reference feature vector $\hat{r}$ is the position estimation.

This position estimation method is also known as the nearest neighbor algorithm. Note that the choice of the distance metric affects the performance [22,23]. In previous work, we found that the $\ell_{1}$-norm performs well in RSS-based device-free localization systems [22].

### 3.3. Feature Vector Composition

After introducing the position estimation method concerning the feature vectors, this section focuses on the construction of the corresponding feature vectors.

A conventional DFL system contains a network of *L* sensor nodes. Here, the corresponding feature vector $s_{p_{r}}$ of the *r*-th position in the target area includes the magnitudes ${\left|h\left(t\right)\right|}_{l}$ of the $L_{S}=\sum_{l=1}^{L-1}l$ links between the *L* nodes, where $L_{S}$ is the number of elements of the feature vector:

(8) $$s_{p_{r}}=\left(\begin{array}{c} {\left|h\left(t\right)\right|}_{1}\\ {\left|h\left(t\right)\right|}_{2}\\ \cdots \\ {\left|h\left(t\right)\right|}_{L_{s}} \end{array}\right).$$

Since several radio-frequency links and the magnitudes are affected by the target, this feature vector characterizes the target positions.

The aim of MAMPI is the reduction of sensor nodes by extracting several multipath components from a single measurement. The application of ultra-wideband communication enables the analysis of multipath propagation effects. As the transmitted signal is reflected on walls and other obstacles, the receiver Rx does not only measure a single link that is affected by the person. With knowledge of the room geometry, we evaluate the influence of the person at multiple components on the received signal.

**Figure 3:**
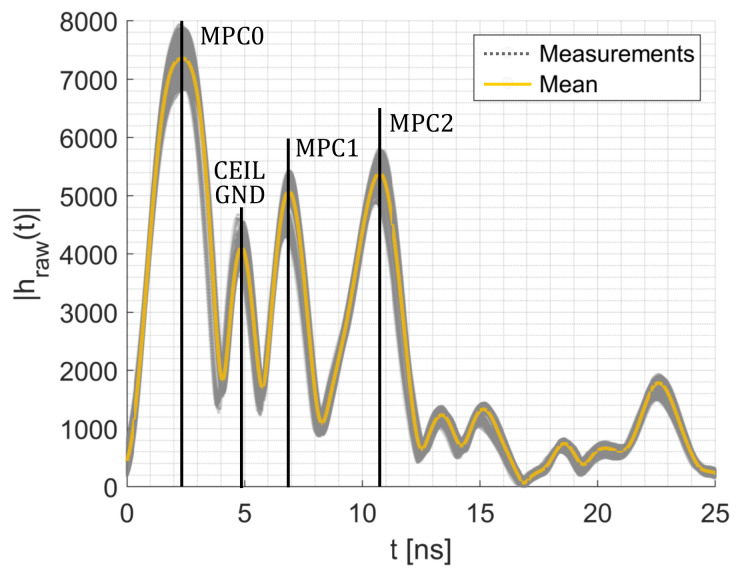
CIR measurements.

Figure 3 shows channel impulse response measurements with several distinguishable multipath components. The first peak indicated as MPC0 is the direct link between the transmitter and the receiver. Next to MPC0, we extract two additional MPCs (indicated as MPC1 and MPC2) that are caused by reflection on the wall from the CIR measurements. With the known positions of the MPCs in the CIR measurement, it is possible to extract the magnitude and the phase of the respective MPCs. In Section 4.2.3, we describe the extraction and processing of the MPCs from the CIR measurements in more detail.

We assume that the following multipath propagation consists of *I* paths. The feature vector $s_{\mathrm{Mag}}$ consists of the magnitude of the *I* multipath components (MPCs) resulting in $L_{S}=I$ elements:

(9) $$s_{\mathrm{Mag}}=\left(\begin{array}{c} {\left|h\left(t\right)\right|}_{\mathrm{MPC}0}\\ \cdots \\ {\left|h\left(t\right)\right|}_{\mathrm{MPC}i}\\ \cdots \\ {\left|h\left(t\right)\right|}_{\mathrm{MPC}(I-1)} \end{array}\right),\;\mathrm{for}\;i=1,...,I-1.$$

In line-of-sight conditions, MPC0 most likely carries the information of the direct link between Tx and Rx and includes an unknown phase offset. To neglect this offset, we calculate the relative phase $\Delta \phi_{i}$ of the *i*-th path with:

(10) $$\Delta \phi_{i}=\phi_{i}-\phi_{0}.$$

The feature vector $s_{\mathrm{Phase}}$ consists of all those phase differences including overall $L_{S}=I-1$ elements:

(11) $$s_{\mathrm{Phase}}=\left(\begin{array}{c} \Delta \phi_{1}\\ \cdots \\ \Delta \phi_{i}\\ \cdots \\ \Delta \phi_{I-1} \end{array}\right)$$

Combining the feature vector $s_{\mathrm{Mag}}$ from Equation (9) and $s_{\mathrm{Phase}}$ from Equation (11) results in feature vector $s_{\mathrm{MPC}}$ consisting of $L_{S}=2I-1$ elements:

(12) $$s_{\mathrm{MPC}}=\left(\begin{array}{c} {\left|h\left(t\right)\right|}_{\mathrm{MPC}0}\\ \cdots \\ {\left|h\left(t\right)\right|}_{\mathrm{MPC}(I-1)}\\ \Delta \phi_{1}\\ \cdots \\ \Delta \phi_{I-1} \end{array}\right).$$

We evaluate those feature vectors in the upcoming sections.

### 3.4. Error Modeling

In the following section, we derive an error model that calculates the position error probability $p_{E}$ for the position estimation introduced in Section 3.2.

**Figure 4:**
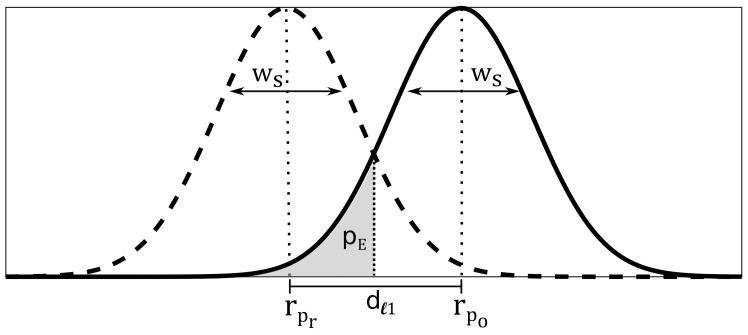
Error model ©2020 IEEE, Reprinted, with permission, from [5].

Figure 4 illustrates our proposed error model. The figure shows the probability density functions (PDFs) of the feature vectors $r_{p_{r}}$ and $r_{p_{o}}$ with $L_{S}$ elements, each. We assume that the probability density functions of the $L_{S}$ elements are normally distributed with the standard deviation of $\sigma_{l}$. The overall noise term $w_{s}$ for one feature vector is calculated as:

(13) $$w_{s}=\frac{2}{3}\sqrt{\sum_{l=1}^{L_{s}}\sigma_{l}^{2}}.$$

Equation (13) calculates the probable error, i.e., half of the values of this error distribution lie inside the interval $\gamma \approx \frac{2}{3}\sigma_{l}$.

The feature vectors $r_{p_{r}}$ and $r_{p_{o}}$ are separated by a distance of $d_{\ell_{1}}\left(r_{p_{o}},r_{p_{r}}\right)$. The position error probability $p_{E}$ describes the probability of estimating the wrong position based on the feature vectors. As illustrated in Figure 4, $p_{E}$ is the overlapping area of the PDFs of $r_{p_{r}}$ and $r_{p_{o}}$. The position error probability $p_{E}$ results to:

(14) $$p_{E}=\frac{1}{2}\mathrm{erfc}\left(\frac{d_{\ell_{1}}\left(r_{p_{o}},r_{p_{r}}\right)/2}{\sqrt{2}w_{s}}\right),$$

with $\mathrm{erfc}\left(x\right)=1-\frac{1}{\pi }\int_{-x}^{x}e^{-t^{2}}dt$ is the complementary error function for normal distributions.

$p_{E}$ depends on the distance between the feature vectors. When the distance between both feature vectors is greater than the noise with $d_{\ell_{1}}\left(r_{p_{o}},r_{p_{r}}\right)\gg w_{s}$, $p_{E}\to 0$. That means that choosing the wrong position is nearly impossible. In the worst-case $d_{\ell_{1}}\left(r_{p_{o}},r_{p_{r}}\right)=0$, the position error probability maximizes with $p_{E}=0.5$ and the position estimation becomes random and the estimation is most unreliably. To minimize $p_{E}$, the choice of elements of the feature vectors that characterize the single positions is important.

## 4. Implementation

In the first part of this section, we describe the simulation setup that is used to compare the performance of the conventional with the multipath-assisted device-free localization system with the proposed position error probability. In the second part of this section, we describe the measurement setup including the signal processing for a real implementation of the system.

### 4.1. Simulation

#### 4.1.1. Raytracing

This section describes how we create feature vectors based on the proposed radio propagation model.

First, we determine the position of the sensors (Tx and Rx), as well as the walls. To reconstruct the wall reflections, we mirror the sensors at the walls to create virtual sensors (see blue dots for the Tx position in Figure 1b). The reflection path results from the link between these virtual transmitters and the real receiver Rx. The intersection of the link with the wall is also the reflection point. The link is as long as the corresponding reflection path. In this paper, we focus on the 1st order reflections. To model higher-order reflections, the virtual positions need to be mirrored at the other walls, respectively.

To simulate our device-free localization systems, we calculate the feature vectors for each position. The feature vector is composed of the different radio links of the setup. The impact on the magnitude is described with Equation (4), and the impact on the phase with Equation (5) respectively. For the conventional DFL system and the 0th-MPC of the multipath-assisted DFL system, we insert the positions of the physical sensors. To calculate the MPCs, we superimpose the radio links of the virtual transmitter $Tx^{\prime }$ and the receiver Rx and the virtual receiver $Rx^{\prime }$ and the transmitter Tx.

In this paper, we neglect the impact of the ceiling and ground reflection. We decided to propose a simple radio propagation model that accounts for most effects on the received signal, while only requiring the diameter of the person. The model accounts for changes in the 2-dimensional space as shown in Figure 2. Therefore, we must ensure that the ground and ceiling reflections do not interfere with the other MPCs. Therefore, we place the sensors as half of the height of the room, which results that the ground and ceiling reflection travel the same distance from the Tx to the Rx, resulting in a single peak in the CIR measurement. To ensure that the other multipath components do not interfere with the unmodeled ground and ceiling reflections, we place the Tx and Rx in a way that the multipath components must travel a larger distance, which places the peaks further away in the CIR measurement. Figure 3 shows the measured CIRs. The ground (GND) and ceiling (CEIL) reflection is placed between MPC0 and MPC1. Another possibility is to include antennas with a radiation pattern that dampens MPCs coming from the ceiling or the ground.

#### 4.1.2. Noise Distributions

For the error model, we assume normally distributed noise for the magnitudes and the phase differences. To prove this assumption the following figures show the histograms and the normal probability density functions of measured magnitude and phase differences of our measurement setup.

**Figure 5:**
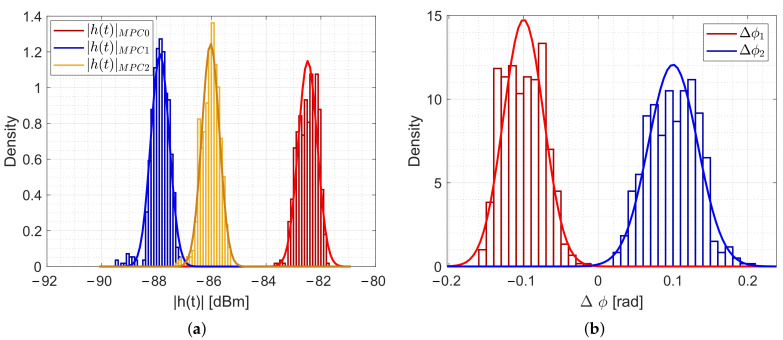
Measured magnitudes and phase differences and the distribution fittings during the idle case. (**a**) PDFs of ${\left|h\left(t\right)\right|}_{\mathrm{MPCs}}$. (**b**) PDFs of Δϕ.

Figure 5 shows the PDFs and the fittings for the magnitudes and phase differences during the idle case that is described in Section 4.2.1.

Figure 5a shows the histogram for the measured magnitudes for the three MPCs. Each multipath component consists of a normal distribution ($\sigma_{{\left|h\left(t\right)\right|}_{\mathrm{MPC}0}}=0.35$ dB, $\sigma_{{\left|h\left(t\right)\right|}_{\mathrm{MPC}1}}=0.34$ dB, $\sigma_{{\left|h\left(t\right)\right|}_{\mathrm{MPC}2}}=0.32$ dB). We conclude to choose $\sigma_{{\left|h\left(t\right)\right|}_{\mathrm{MPC}}}=0.35$ dB as the noise term for the magnitudes.

Figure 5b shows the measured phase differences. To differentiate the distributions in the same figure, we shifted the mean to $\mu_{\Delta \phi_{1}}=-0.1$ and $\mu_{\Delta \phi_{2}}=0.1$. The fitting shows that the phase differences are normally distributed. For the phase differences, we determine $\sigma_{\Delta \phi {}_{1}}=0.027$ rad and $\sigma_{\Delta \phi {}_{2}}=0.033$ rad. For the simulations, we will choose $\sigma_{\Delta \phi }=0.035$ rad as the noise term for the phase differences.

#### 4.1.3. Description of the Algorithm

With our simulation setup, we aim to assess whether the multipath-assisted DFL system provides similar performance to a conventional DFL system. Furthermore, we determine the impact of the different feature vectors on the proposed error probability $p_{E}$. To assess whether the assumptions for our analytical model are valid, we implement a Monte-Carlo simulation. Algorithm 1 shows the pseudo-code of the algorithm that calculates the position error probability with the analytical model and via Monte-Carlo simulation.

**Algorithm 1:** Pseudo-code for the error modeling and Monte-Carlo simulation ©2020 IEEE, Reprinted, with permission, from [5].

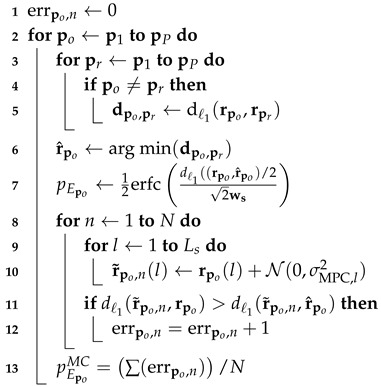



After initialization, we calculate the $\ell_{1}$-distance for the feature vector of the current observation $p_{o}$ to the feature vectors $p_{r}$ of all other positions, where $p_{o}\neq p_{r}$ (line 2–5). In Line 6, we determine the feature vector ${\hat{r}}_{p_{o}}$ that inherits the smallest distance. Based on this distance, we determine the position error probability $p_{E_{p_{o}}}$ for that position. The Monte-Carlo simulation is shown in Line 8–13: First, we add normally distributed noise to every element of our feature vector (Line 9–10). Then we determine, whether the distance of our noisy feature vector ${\tilde{r}}_{p_{o},n}$ compared to our observation $r_{p_{o}}$ is larger than the distance of the noisy feature vector ${\tilde{r}}_{p_{o},n}$ to our estimation ${\hat{r}}_{p_{o}}$. If so, we detected an erroneous position estimation due to the noise of the feature vectors. In Line 13, we calculate the error probability for each position by summing up the number of errors and dividing it by the number of Monte-Carlo simulations *N*.

#### 4.1.4. Simulation Setup

We aim to deploy our multipath-assisted device-free localization system in a corridor similar to Figure 1b. In the simulation, we assume a 5 × 5 m corridor with two walls (y=0 and y=5) that create reflections. We place the transmitter Tx at (0,2.5) and the receiver Rx at (5,2). This asymmetrical geometry prevents that the simulated MPCs becomes identical, leading to multiple minima in the nearest neighbor algorithm. Another goal of the sensor placement is that the MPCs be at least 1 ns apart from each other so that the multipath components extracted from DW1000 CIR measurements do not overlap. In the simulation, we assume the center frequency of the radio chip as 4.4928 GHz with a bandwidth of 499.2 MHz, which corresponds to IEEE 802.15.4.a Channel 3 [24]. We assume the diameter of the person b=0.28 m and calculate the positions with a spacing of 0.2 m. The diameter of the person was empirically determined with 0.28 m. As described in Section 4.2.1, the length of the person is approx. 0.45 m from shoulder to shoulder and 0.25 m from chest to the back. Therefore, the diameter of the simulation must be a value between 0.25 m and 0.45 m. Due to the orientation of the person during the measurements the affected area is close to the length between the back and the chest. We perform N=1500 Monte-Carlo simulations and calculate the empirical cumulative distribution function (ECDF) of $p_{E}$ for each system. To compare the multipath-assisted DFL system with the conventional DFL system, we place the sensors at the same Tx and Rx positions and at the reflection points on the walls.

### 4.2. Measurements

In this section, we provide the details of our measurement setup, hardware, and the signal processing that is necessary to extract the multipath components from the measured CIR.

#### 4.2.1. Measurement Setup

**Figure 6:**
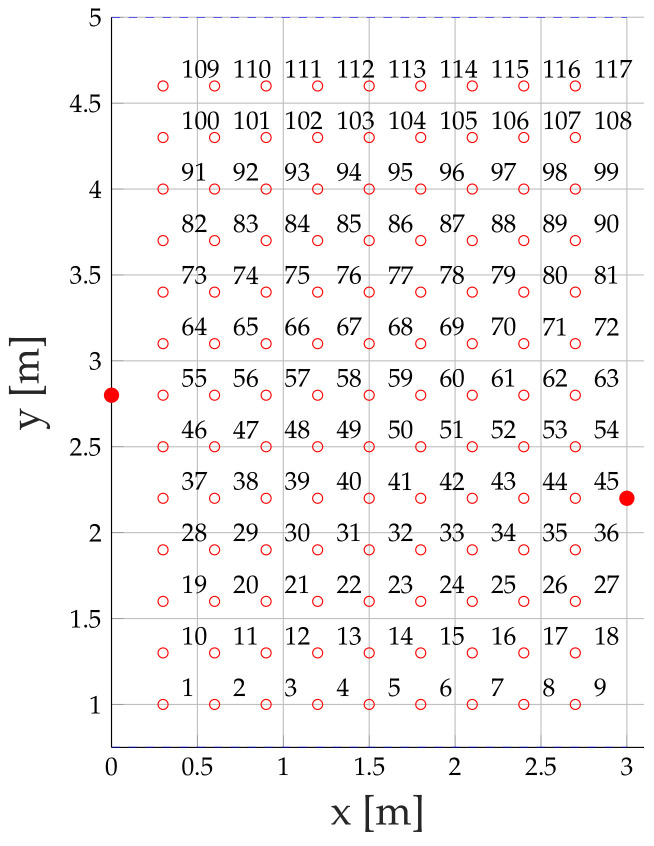
Measurement setup.

Figure 6 shows the measurement setup that covers the target area within a corridor of approx. 3 × 4 m.

In the measurement setup, we place the transmitter Tx at (0,2.8) and the receiver Rx at (3,2.2). The signal is reflected at y=0.75 and at y=5. To cover the target area of this corridor, we select an equal spacing of 0.3 m between every position. We keep a distance of at least 0.25 m towards the sensors, and the walls to prevent the person to contact a wall or a sensor. This results in x-position starting at x=0.3 and ending at x=2.7, and in y-direction starting at y=1 and ending at y=4.7. The height of the corridor is 2.54 m. We place the Tx and Rx at half of the height of the corridor resulting in h=1.27 m. The antennas of our Tiny TriSOS nodes face each other during the measurements.

We decided to choose a section of a corridor because it is a reproducible scenario that creates sufficient data to provide a proof-of-concept for our proposed system. We require that we have at least three MPCs that can be distinguished from CIR measurements. Furthermore, the measurement grid must be fine enough that the effect of the person can be seen in the measurements and the model. Currently, the measurement setup contains 117 positions that we try to localize with feature vectors composed of 2 to 5 elements. Deploying a finer grid might require a more advanced localization algorithm, which is part of the future work. In the future, we want to set up the system solely based on the geometry and idle measurements. Therefore, we will increase the target area and focus on different challenges, such as different wall materials and overlapping of MPCs resulting in constructive and destructive interference.

Our measurement equipment based on the Decawave DW1000 measures the CIR with a resolution of 1 ns. Therefore, we ensure that the multipath components are placed at least 1 ns apart from each other. We calculate the positions of the multipath components as the distances from the (virtual) Tx towards the Rx:

(15) $$t_{\mathrm{MPC}}=\frac{||Tx-{Rx||}^{2}}{c_{0}},$$

where $c_{0}$ is the speed of light and $\left|\right|\cdot {\left|\right|}^{2}$ the Euclidean distance.

**Figure 7:**
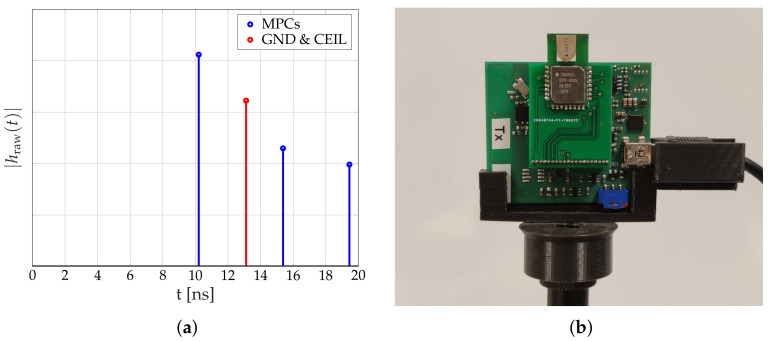
Theoretical multipath components and measurement equipment. (**a**) Estimated positions of the MPCs. (**b**) Photograph of the Measurement Equipment.

Figure 7a shows the calculated MPCs based on the measurement setup. After transmission, the 0th-MPC will arrive after 10.21 ns, followed by the ground and ceiling reflection at 13.11 ns. The 1st-MPC will arrive after 15.38 ns, and the 2nd MPC after 19.45 ns.

At the start of the measurements, we record 600 idle CIR measurements, while the target area is vacant. We will apply those idle values to confirm the positions of the multipath components and to calibrate the magnitudes as well as the phase differences. For each of the 117 positions shown in Figure 6, we record 125 CIR measurements while the person is standing at the respective position. The person has a height of 1.95 m, a length from chest to the back of approx. 0.25 m and a length from the left to the right shoulder of approx. 0.45 m. As described before, our radio propagation model calculates the alteration of the persons’ position in a 2-dimensional space neglecting the height of the person. The sensors are mounted at a height of 1.27 m, which is at the upper-body level of the person. The person in the target area is facing south for the duration of the measurements.

We provide the measurements in the Supplementary Materials.

#### 4.2.2. Measurement Equipment

We implement the UWB CIR measurements with our evaluation hardware called Tiny TriSOS. The Tiny TriSOS is a wireless sensor node that we developed in former projects and is the same hardware that we choose for time-of-flight indoor localization applications [25]. The Tiny TriSOS features an 8-bit microcontroller the ATXmega128 from Atmel and is equipped with the DWM1000 from Decawave that features the DW1000 from Decawave as a UWB radio chip.

Figure 7b shows the Tiny TriSOS mounted on a microphone stand.

In the following, we describe the settings of the UWB transceiver. For the measurements, we take IEEE 802.15.4a channel 3, with a center frequency $f_{c}=4.4928$ GHz [24]. The bandwidth of the signal is B=499.2 MHz, and the pulse repetition frequency PRF=64 MHz. We choose a preamble length of 128, the preamble acquisition chunk size of 8, *Tx* and *Rx* preamble code of 9, and a data rate of 6.8 MBit/s. Next to the measurement of the CIR, the DW1000 from Decawave enables us to perform a simple two-way ranging to determine the distance between the transmitter and receiver [25]. Measuring the distances between the sensors can enable automatic anchor self-localization in the future [26]. With our measurement equipment, we measure the CIR based on 179 I/Q values with approx. 9 Hz. We provide the measurements in the Supplementary Materials.

#### 4.2.3. Extraction and Processing of the CIR Measurements

This section describes the process of converting the Decawave DW1000 CIR measurements into magnitude and phase-difference values of the respective multipath components.

**Figure 8:**
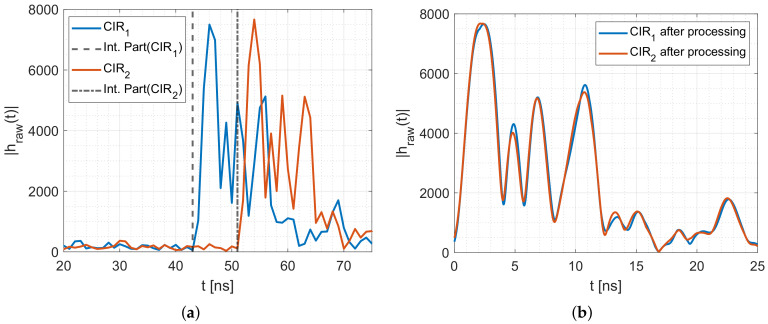
Measured and aligned CIR measurements. (**a**) Two consecutive CIR measurements. (**b**) CIRs measurements after alignment.

Each CIR $h_{\mathrm{raw}}\left(t\right)$ is a series of 179 complex I/Q values. The Decawave DW1000 measures the CIR with approx. 1 ns between each sample. From the I and Q values, we calculate the magnitude $|h_{\mathrm{raw}}\left(t\right)|$. Figure 8a shows the magnitude of two consecutive CIR measurements during the idle phase. Each CIR shows four distinctive peaks in the magnitudes. The first peak (MPC0) is the direct path from the Tx to the Rx. The first peak of the blue CIR is at approx. 46 ns, the second at approx. 49 ns, the third (MPC1) at approx. 52 ns and the fourth (MPC2) at approx. 56 ns. Based on the register value of the DW1000, the blue CIR starts at 43 ns (dashed line). The orange CIR is the consecutive measurement starting at 51 ns (dashed-dotted line) but shows the same relative behavior and peaks.

Because consecutive CIR measurements do not necessarily start at the same time, we need to align the CIR measurements as shown in Figure 8. The Decawave DW1000 applies an internal leading-edge algorithm that provides the time stamp of the rise of the 0-th multipath component MPC0 as a combination of an integer part and a fractional part [27]. Those values are shown by the dashed lines in Figure 8a. To align each CIR measurement, we shift the time axis to be zero at the respective timestamp of its rising edge, which synchronizes the beginning of the single CIR measurements.

Additionally, we apply a sinc-interpolation to the measurements. We decided to apply a sinc-interpolation because the spectrum of the IEEE 802.15.4a channel 3 is nearly rectangular. For the sinc-function, we double the bandwidth to 2B=1 GHz to avoid unwanted interference between the adjacent I and Q samples. To fit the CIRs, we formed the sinc-function with sample time $\Delta t_{sinc}\ll 1/\left(2B\right)$. We add zeros between the samples of the CIR to ensure the same sample time for the fitting and measurement. Afterward, we convolve the sinc-function with the expanded CIR measurements. Figure 8b shows the interpolated and aligned CIR measurements.

The fitting is carried out for the comparability of the CIR measurements and to reconstruct the CIR that has been sampled by the DW1000 with 1 ns. The Decawave DW1000 provides the following equation to determine the magnitude of the first peak $P_{FP}$, which is the magnitude of MPC0:

(16) $$P_{FP}=10\;\log_{10}\left(\frac{F_{1}^{2}+F_{2}^{2}+F_{3}^{2}}{N^{2}}\right)-A\;\left[\mathrm{dBm}\right],$$

where $F_{1}$, $F_{2}$, and $F_{3}$ are the magnitude values reported by the leading-edge algorithm of the DW1000. Here, *N* is the preamble accumulation count, and the constant A=121.74 dB is chosen for a pulse repetition frequency of 64 MHz [28].

In general, conventional DFL systems analyze the magnitude of MPC0 or the overall received power level $P_{Rx}$. Our multipath-assisted DFL system analyzes the magnitude and phase of all MPCs that are measured by the CIR. In [6] we have shown that this approach is applicable for arbitrary multipath components. Therefore, we determine the measured magnitudes of $|h_{raw}\left(t\right)|$ of MPC1 and MPC2.

After calculating the magnitude of each multipath component, we need to scale those values in terms of the receive power level $P_{Rx}$ of the measured CIR. To estimate the received power $P_{Rx}$, the DW1000 records the CIR power value *C* of the measurement [28]. $P_{Rx}$ is calculated as follows:

(17) $$P_{Rx}=10\;\log_{10}\left(\frac{C\cdot 2^{17}}{N^{2}}\right)-A\;\left[\mathrm{dBm}\right],$$

where constant A=121.74 dB chosen as above [28].

To scale the measured CIRs we calculate the mean of the $P_{Rx}$ of all idle measurements ${\bar{P}}_{Rx,idle,MPC}$. Then, we subtract the receive power level $P_{Rx}$ of the current measurement from the corresponding ${\bar{P}}_{Rx,idle,MPC}$:

(18) $$\Delta P_{Rx}={\bar{P}}_{Rx,idle,MPC}-P_{Rx,MPC}\;\left[\mathrm{dB}\right]$$

#### 4.2.4. Processing of the Phase

To extract the phase of the CIR measurements, we determine the complex values at the respective MPCs.

**Figure 9:**
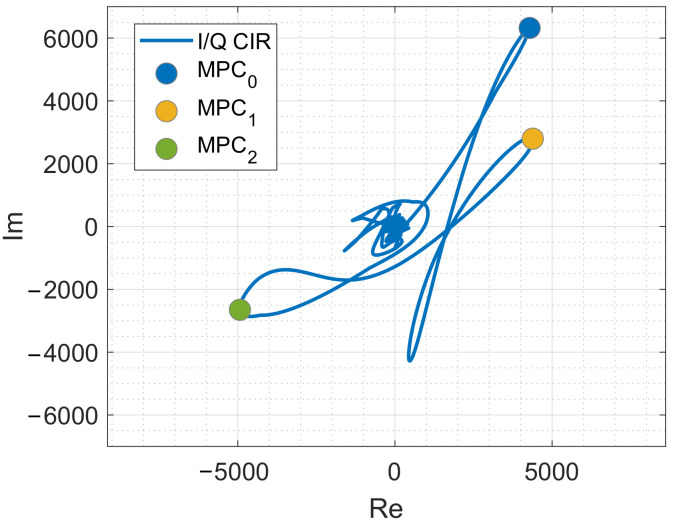
Complex CIR measurement with values of the multipath components.

Figure 9 shows the measured CIR of Figure 8b in the complex plane. The CIR starts close to (0,0), the complex value of MPC0 is at the blue dot, followed by the value of the MPC1 (yellow dot) and the value of MPC2 (green dot). The peaks in the magnitude are the loops in the complex plane. The ground and ceiling reflection is the loop that is not marked explicitly in the figure.

Inspecting the complex CIR might help in the future to determine the number of peaks and to localize them accurately.

To receive the corresponding phases of the MPCs, we calculate the phase of the reconstructed and aligned CIRs with the following equation

(19) $$\phi =\arctan\left(\frac{I\{h(t)\}}{R\{h(t)\}}\right)\;\left[\mathrm{rad}\right].$$

Then we unwrap ϕ and read the phases at the corresponding MPC positions. Finally, we calculate the relative phases $\Delta \phi_{i}$ using Equation (10).

## 5. Evaluation

In the first part of the section, we compare the conventional DFL system with our proposed multipath-assisted DFL system. Furthermore, we compare the analytical model of the position error probability to the result achieved by the Monte-Carlo simulation. In the second part, we compare our radio propagation model with measurements done in an indoor scenario. In the last part of this section, we set up the multipath-assisted DFL system in an indoor scenario and compare the localization results to our simulated position error probabilities.

### 5.1. Simulation of the Position Error Probability

**Figure 10:**
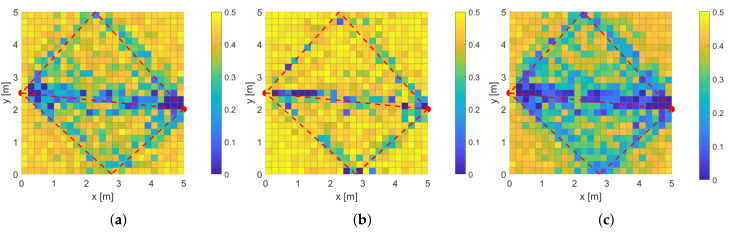
Simulated position error probability $p_{E}$ of the multipath-assisted DFL system with different feature vectors. The color coding represents $p_{E}$. (**a**) Magnitudes only. (**b**) Phase differences only. (**c**) Combination of magnitudes and phase differences.

**Figure 11:**
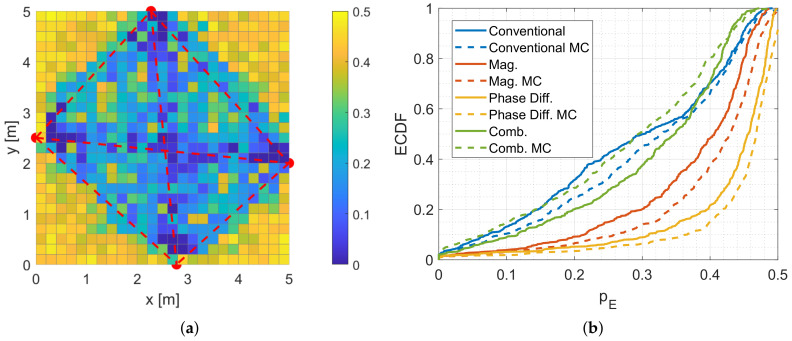
Simulation results for the different DFL systems. (**a**) Simulation of the conventional DFL system. The color coding represents $p_{E}$. (**b**) Comparison of the position error probabilities of the simulated systems.

In the following, we will compare the multipath-assisted DFL system with the conventional DFL system using the proposed position error probability $p_{E}$. Figure 10 shows the results of the multipath-assisted device-free localization system for the different feature vectors. Figure 11a shows the results of the conventional device-free localization system based on the magnitudes of the direct radio-frequency links. When $p_{E}$ has a yellow color, the position error probability is high, meaning that the position cannot be localized reliably. When $p_{E}$ has a blue color, the position error probability is low, therefore, the position estimation will be accurate. In the figures, we indicate the physical sensor positions by a red dot and for orientation, we show the radio links of the system as red dashed lines.

For the multipath-assisted DFL system, we compare the same setup, but with different feature vectors.

Figure 10a shows the position error probability based on the feature vector $s_{\mathrm{Mag}}$ of the magnitudes of the $L_{S}=3$ MPCs. Close to the sensor positions and between the direct link between Tx and Rx, the position error probability is low. The corners of the rooms and the areas between the reflections and the direct link cannot be localized accurately.

Figure 10b shows the position error probability based on the phase differences of the three MPCs. The three MPCs result in a feature vector $s_{\mathrm{Phase}}$ that is composed of two-phase differences ($L_{S}=2$). The area that is covered by the system is smaller, only a small trident close to the sensors inherit a low position error probability.

Figure 10c shows the position error probability based on the combination of the magnitudes and phase differences of the three MPCs. This results in a feature vector $s_{\mathrm{MPC}}$ based on $L_{S}=5$ values. The combined feature vector enables a low position error probability at positions that are close to the links. The overall position error probability is lower than the feature vector based on the magnitude and phase difference only. This indicates that a larger feature vector improves the localization system, as long as the new vector elements have similar noise terms.

We compare the multipath-assisted DFL system (Figure 10) with a conventional DFL system shown in Figure 11a. For a better comparison, we choose the same scenario and deploy the sensors at the same positions as the multipath-assisted system and the point of the reflections. The conventional DFL system is based on four physical sensors, which results in $L_{S}=6$ radio-frequency links. Similar to the multipath-assisted systems, the position error probability at the corners is very high due to a lack of coverage. Close the sensors, and at the intersections of the links, the position error probability is low. Overall, the area that is covered by the links can be localized.

To evaluate the different DFL systems, we calculate the empirical distribution functions (ECDFs) of the different systems. Figure 11b shows the results. The solid lines are the position error probability $p_{E}$ of the analytical model, the dashed lines are the position error probabilities $p_{E}^{MC}$ calculated by the Monte-Carlo simulations. The position error probability of the system composed of phase differences is higher than the other feature vectors. The system based on the magnitudes is slightly better. The system based on the combination of magnitude and phase differences performs close to the conventional DFL system, demonstrating the possibility to reduce the number of the required sensors by employing MPCs. The Monte-Carlo simulations of the phase difference, magnitude, and conventional system predict a slightly higher position error probability, although the curvature stays similar. The Monte-Carlo simulation for the combined feature vectors shows the same curvature as the analytical model but overestimates the result. Despite the deviations, we conclude that the analytical model provides an estimation of the expected performance of the DFL system.

The simulations demonstrate that the performance of the multipath-assisted device-free localization system based on two physical sensors is close to a conventional one based on four physical sensors. However, we need to employ the feature vector with more elements to achieve similar accuracy.

### 5.2. Comparison of the Model with the Reference Measurements

In this section, we compare our radio propagation model with measurements. Ideally, the model performs well enough to replace the training phase of the fingerprinting in a future system.

**Figure 12:**
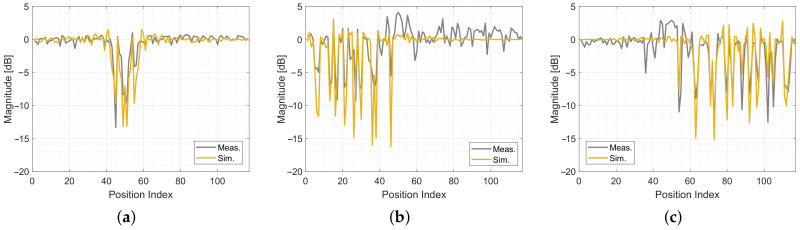
Measured and simulated magnitudes. (**a**) Simulated and measured ${\left|h\left(t\right)\right|}_{\mathrm{MPC}0}$. (**b**) Simulated and measured ${\left|h\left(t\right)\right|}_{\mathrm{MPC}1}$. (**c**) Simulated and measured ${\left|h\left(t\right)\right|}_{\mathrm{MPC}2}$.

Figure 12 shows the simulated and measured magnitudes at each reference position. The yellow lines are the simulated values of the radio propagation model. The gray lines are the mean of the measurements of each position. To compare the model to the measurements, we calculate the mean of the respective magnitudes and phase differences for every MPC and every position.

In Figure 12, we show the magnitudes of the three MPCs. The different positions are shown on the x-axis in Figure 6. Approx. the first third of the positions are covered by the reflection at the lower wall. The second third covers the direct radio link between the Tx and the Rx. The last third covers the reflection on the upper wall. Figure 12a shows the magnitude of the direct link. When the person is at a position close to the link, the magnitude drops up to 15 dB. Outside the direct link, the effect of the magnitude is close to zero. Overall, the model fits very well with the measurements. The magnitude of the lower reflection is shown in Figure 12b. When the person stands at a position close to the link, the magnitude drops up to 10 dB. In the first third of the positions, the model can predict the changes of the magnitude well, in the last two thirds the measurements show some oscillations that are not predicted by the model. Figure 12c shows the magnitude of the upper reflection. As for the first reflection, the magnitude drops up to 10 dB, when the person is close to the link. The model and the measurements predict this behavior well.

**Figure 13:**
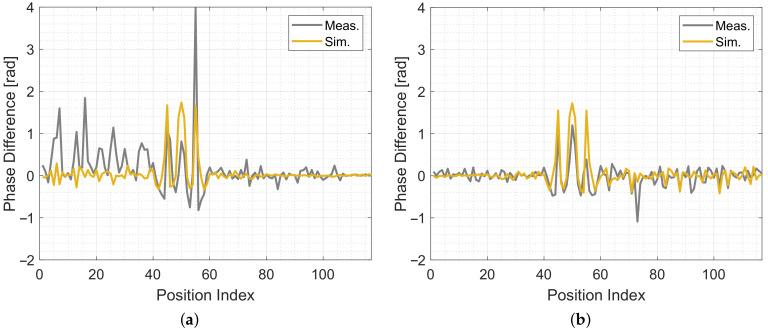
Measured and simulated phase differences. (**a**) Simulated and measured $\Delta \phi_{1}$. (**b**) Simulated and measured $\Delta \phi_{2}$.

Figure 13 shows the simulated and measured phase differences at each reference position. Similar to the magnitudes shown in Figure 12. On the x-axis are the positions of the measurement setup shown in Figure 6. $\Delta \phi_{1}$ (Figure 13a) and $\Delta \phi_{2}$ (Figure 13b), are very similar to each other. As expected, the change in the phase difference is large, when the person is close to the direct link between Tx and Rx. There are only small phase-difference changes when the person is close to a reflection. $\Delta \phi_{1}$ has more variation at the first third of the positions and $\Delta \phi_{2}$ more in the last third of the positions. Especially for $\Delta \phi_{1}$, we see phase-wrapping errors, which is an issue that we need to solve in the future. Still, our model can predict the change of the phases.

In the future, we investigate, whether the performance of the model is good enough to replace the training phase. The proposed model only requires a few inputs (Tx, Rx, the position of walls, and the position and diameter of the person) and can predict the magnitude and phase. Calibration of the values is made solely with idle measurements that are recorded while the target area is vacant and is easily repeated, e.g., at night.

### 5.3. Evaluation of the Measurement Setup

In this section, we present the measurement results of our system together with the simulated position error probabilities.

**Figure 14:**
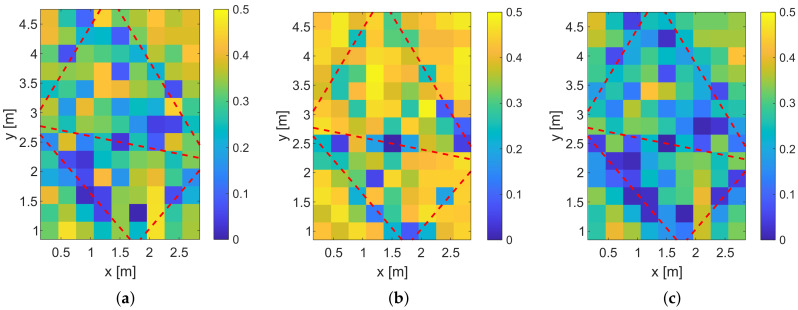
Simulation of $p_{E}$ of the measurement setup. (**a**) Simulated $p_{E}$ for magnitude only. (**b**) Simulated $p_{E}$ for phase differences only. (**c**) Simulated $p_{E}$ for combination of magnitude and phase differences.

In a first step, we calculate the position error probabilities $p_{E}$ of our measurement setup shown in Figure 6. Figure 14 shows the results.

The position error probabilities are color-coded, where yellow means that the $p_{E}$ is very high and dark blue that $p_{E}$ is very low. The red dashed lines show the transmission paths of the MPCs. Similar to the previous simulation, the system using a feature vector based on the magnitudes or phase differences only provides higher position error probabilities than the combination of both feature vectors. The simulated position error probabilities show that we expect good localization results close to the direct link between Tx and Rx and near the reflections.

To compare the simulations with real measurements, we implement the same fingerprinting algorithm described in Section 3.2 as for the simulations. We divided the reference data composed of 125 CIR measurements at each position into a training and a test data set, which is a common practice in machine learning applications [29]. Instead of using 80% of the data for training and the other 20% for validation, we chose a training data set with approx. 10% of the 125 measurements, resulting in 13 training CIR measurements and 112 evaluation CIR measurements. As the reference fingerprints, we used the mean of the training set for each position, and as the test set, we used the evaluation values. In the future, we aim to replace the fingerprinting data set with our model and idle measurements, which is an outlook for future work. The intention is that we avoid the deterioration of the fingerprints over time as shown in [30], by calibrating the system automatically.

For each position, we determined the relative incidence of localization errors: When the observation of the test set leads to a different position than its original position, we count a localization error. We calculate the relative incidence of localization errors as the number of false estimations over the number of observations. Therefore, the relative incidence of localization errors ranges from 0 to 1, where 0 means that every observation from the evaluation set was correct. And 1 means that every observation was incorrect. In addition to the calculation of the relative incidence of localization errors, we determine the localization error. We define the localization error as the Euclidean distance between the estimated position and the ground truth position.

**Figure 15:**
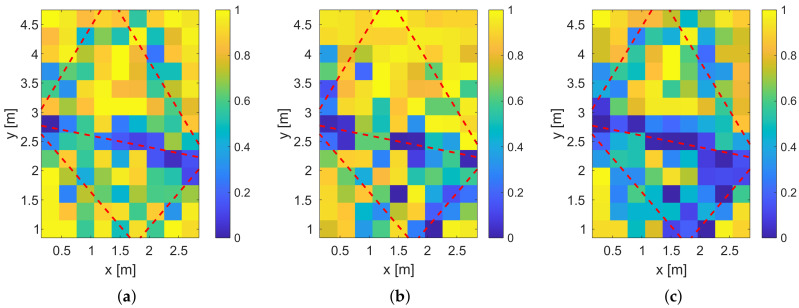
Fingerprinting results. (**a**) Measured relative incidence of localization errors for magnitude only. (**b**) Measured relative incidence of localization errors for phase difference only. (**c**) Measured relative incidence of localization errors for the combined magnitude and phase differences.

Figure 15 shows the result of the fingerprinting for the different feature vectors. All three systems have in common that the lower-left corner and the area between the direct link and the upper reflection are not localized reliably, which is covered by the simulation results. Also, for all three systems, the positions close to the direct links provide a low relative incidence of localization errors. The results of the magnitudes and the phase differences are very similar. In contrast to the simulations, the system that is based on phase differences provides a lower incidence of localization errors than the magnitude only system. We expect that this is caused by the phase wrapping of the measurements that help in the differentiation of the positions. As for the simulations, the combination of the magnitudes and phase differences provides the best result.

Similar to the simulations, we will compare the results of the simulation and the fingerprinting with the empirical distribution functions (ECDFs).

**Figure 16:**
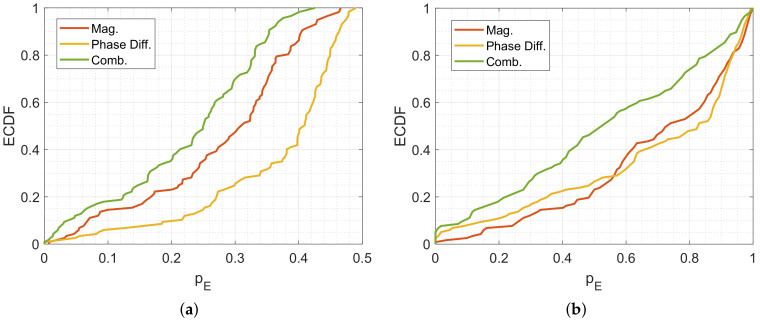
ECDFs for the simulated and measured error probability. (**a**) $p_{E}$ determined by simulation. (**b**) Relative incidence of localization error determined by fingerprinting.

Figure 16a shows the ECDF of the simulated $p_{E}$. To compare the different systems numerically, we take a closer look at the percentage of the positions that have a position error probability lower than 0.25. For the systems that include only the phase differences $p_{E}\left(0.25\right)$ is at approx. 15%, For the systems that include only the magnitudes $p_{E}\left(0.25\right)$ is at approx. 35%, and for the systems that include the combination as a feature vector it is at approx. 50%.

To compare those numbers with our measurement setup, we evaluate the ECDF at the measured relative incidence of localization error of 0.5. For the magnitude only system, the ECDF is at approx. 23%, and for the phase difference only system it is at approx. 27%. The combination results in a value of approx. 48%. This means that the system with the combined feature vectors results in a similar accuracy compared to the simulations.

**Figure 17:**
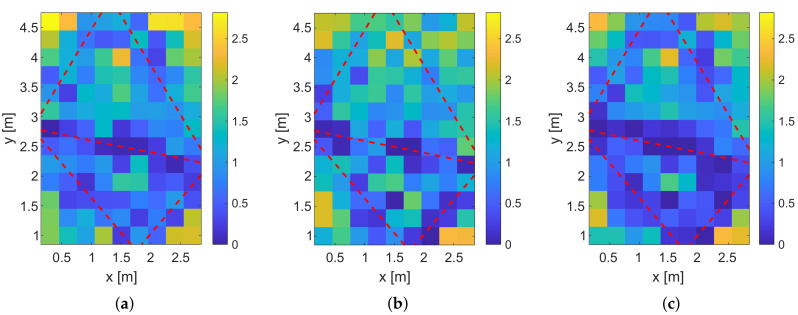
Mean localization error for the fingerprinting. (**a**) Measured mean of the localization error for magnitude only. (**b**) Measured mean of the localization error for phase difference only. (**c**) Measured mean of the localization error for the combined magnitude and phase differences.

In Figure 17, we provide the localization error for the different feature vectors.

By comparing Figure 17a–c, the localization error close to the radio links is low. The corners of the target area cannot be localized reliably. Furthermore, we see that the areas in between the MPC0 and the other MPCs provide high localization errors because the person does not alternate the radio-frequency propagation as much as in direct proximity of the radio links.

**Figure 18:**
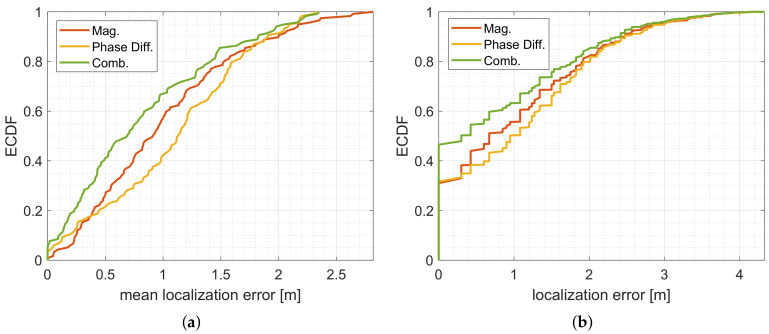
ECDFs for the measured localization error. (**a**) Mean of localization error determined by fingerprinting. (**b**) Localization error determined by fingerprinting.

To compare the results of Figure 17 numerically, we provide the ECDFs of the mean of the localization error in Figure 18a.

Figure 18b shows the ECDFs of all position estimates (117 positions × 112 measurements of the test set). For the feature vectors based on the magnitude and phase differences only, we achieve a localization error of 0 m in approx. 31% of the measurements. For the combined feature vectors approx. 47% of the measurements contain a localization error of 0 m. The 0.8-percentile is 2.0 m, 1.9 m, and 1.8 m for the system based on phase differences, magnitude, and a combination of both. Qualitatively, the localization error confirms the results of our previous investigations. At positions that inherit a low relative incidence of localization error, the localization error is also low. Especially in proximity to the radio links, the systems localize a person reliably.

In this section, we set up a DFL system with several MPCs that are extracted from UWB CIR measurements. Using a simple fingerprinting approach, we achieved similar performance to the simulation based on an analytical model. Furthermore, at positions that inherit a low position error probability, the localization error is also low. However, to fully cover a target area, we need to design the paths of the MPCs carefully. Additional MPCs may be created by increasing the number of sensor nodes, or by using higher-order MPCs. To increase the size of the feature vectors, the UWB sensor nodes may measure the CIR measurement over several frequencies.

## 6. Conclusions and Future Work

In this paper, we developed MAMPI, a multipath-assisted DFL system that is based on magnitude and phase measurements from two sensor nodes that measure the UWB channel impulse response. Although conventional DFL systems need to deploy a larger number of nodes to extend the coverage of the target area, the multipath-assisted DFL system extracts multipath components of the channel impulse response. We proposed an analytical model for the calculation of the position error probability and compared the performance of different feature vectors to a conventional DFL system. Simulations and measurements lead to the conclusion that the combination of magnitude and phase differences leads to a lower position error probability and a lower localization error.

In the future, we want to increase the performance of the system. To do so, we aim to increase the size of the feature vector, e.g., by deploying an additional sensor node, by using multiple different frequencies for measuring the CIR, or by using multipath components of a higher order. In addition, by applying the position error probability, we might limit the positions within the target area in a way that all the feature vectors are distinguishable from each other. Furthermore, we will increase the size of the target area and determine how different wall materials and other obstacles such as furniture impact the system.

## Supplementary Materials

The materials are available online at http://www.mdpi.com/1424-8220/20/24/7090/s1.

## Abbreviations

The following abbreviations are used in this manuscript:

CSI	channel state information
CIR	channel impulse response
DFL	device-free localization
ECDF	empirical cumulative distribution function
MAMPI	multipath-assisted device-free localization with magnitude and phase information
MC	Monte-Carlo
MPC	multipath component
PDF	probability density function
PRF	pulse repetition frequency
RF	radio frequency
RSS	received signal strength
UWB	ultra-wideband
